# Cell-free reconstitution of peroxisomal matrix protein import using *Xenopus* egg extract

**DOI:** 10.1016/j.xpro.2023.102111

**Published:** 2023-02-11

**Authors:** Michael L. Skowyra, Tom A. Rapoport

**Affiliations:** 1 Howard Hughes Medical Institute and Department of Cell Biology, Harvard Medical School, 240 Longwood Avenue, Boston, MA 02115, USA

**Keywords:** Cell Biology, Cell separation/fractionation, Metabolism, Microscopy, Model Organisms, Molecular Biology, Molecular/Chemical Probes

## Abstract

Peroxisomes are vital metabolic organelles whose matrix enzymes are imported from the cytosol in a folded state by the soluble receptor PEX5. The import mechanism has been challenging to decipher because of the lack of suitable *in vitro* systems. Here, we present a protocol for reconstituting matrix protein import using *Xenopus* egg extract. We describe how extract is prepared, how to replace endogenous PEX5 with recombinant versions, and how to perform and interpret a peroxisomal import reaction using a fluorescent cargo.

For complete details on the use and execution of this protocol, please refer to Skowyra and Rapoport (2022).^1^

## Highlights

• Efficient protein import into peroxisomes recapitulated with *Xenopus* egg extract
• Gentle procedure for preparing egg extract that preserves peroxisomal integrity
• Quantitative fluorescence imaging of multiple import cycles mediated by the receptor PEX5
• Efficient depletion of PEX5 from egg extract and re-addition of recombinant versions

## Abstract (editor highlights)

Publisher’s note: Undertaking any experimental protocol requires adherence to local institutional guidelines for laboratory safety and ethics.

## Graphical abstract

**Figure:**
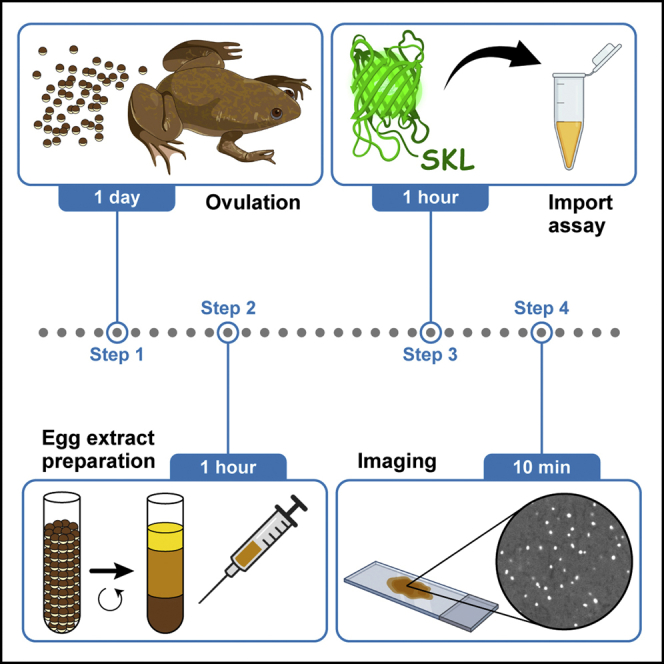

## Before you begin

Peroxisomes occur in nearly all eukaryotic cells^2^ and perform important functions related to lipid metabolism^3^ and redox homeostasis,^4^ among others.^5^^,^^6^^,^^7^ Peroxisomes are vital for human health, as defective import of enzymes into the peroxisomal lumen (matrix) causes severe neurological disorders.^8^ Without exception, peroxisomes import their matrix proteins from the cytosol.^9^ Proteins can be imported into peroxisomes in a folded state or even as oligomers,^10^ which fundamentally differs from protein import into the endoplasmic reticulum or mitochondria.^11^^,^^12^ Understanding how proteins are imported into peroxisomes is thus of considerable therapeutic and scientific interest.

Most peroxisomal matrix proteins contain a C-terminal peroxisome targeting signal (PTS) that consists of the amino acid sequence Ser-Lys-Leu (SKL) or variants of it.^13^ The PTS is recognized in the cytosol by the soluble receptor PEX5.^14^ PEX5 is recruited to peroxisomes by the membrane proteins PEX13 and PEX14, and then moves the cargo into the matrix.^9^ PEX5 must then return to the cytosol. This retrieval process requires PEX5 to be ubiquitinated on a conserved cysteine^15^^,^^16^ by the PEX2-PEX10-PEX12 ubiquitin ligase complex^17^ and extracted into the cytosol by the PEX1-PEX6 AAA ATPase.^18^ Deubiquitination in the cytosol allows PEX5 to start another round of import.^19^^,^^20^ While this overall pathway of peroxisomal import is well established, the underlying mechanism is poorly understood.

*In vitro* systems have provided invaluable insight into the import mechanism.^21^^,^^22^ One type of import system uses semi-permeabilized cells that are bathed in a cytosolic extract containing reporter cargo and import components.^23^^,^^24^^,^^25^ Another uses post-nuclear supernatants or purified peroxisomes prepared from cells^26^^,^^27^ or from tissues such as animal liver^28^^,^^29^ or plant cotyledons.^30^^,^^31^ Post-nuclear supernatants are particularly attractive because they can be easily manipulated or scaled up.^28^ However, current import systems have several disadvantages. The procedures for permeabilizing cells or homogenizing tissues must be carefully optimized to avoid damaging peroxisomes,^25^^,^^28^^,^^32^ which are notoriously fragile.^33^ Cytosolic extracts and post-nuclear supernatants are also inevitably diluted,^23^^,^^28^ so components are not present at physiological concentrations. Crucially, current assays do not allow multiple rounds of protein import,^34^^,^^35^ and lack methods for depleting import components.

To overcome these limitations, we have recapitulated peroxisomal import using *Xenopus* egg extract.^36^ Extract corresponds to the cytoplasm of eggs from the African clawed frog, *Xenopus laevis*. It is prepared by a quick and gentle procedure that preserves organellar integrity and maintains cytosolic components near their physiological concentrations.^37^ Import can be quantitatively followed in extract over time using fluorescent proteins fused to a PTS. Crucially, extract supports multiple import cycles^1^ and a constant import rate over several hours.^36^ PEX5 and other soluble components can be acutely and efficiently depleted from extract and replaced with mutant versions, minimizing indirect effects that might arise in cells. This cell-free system has allowed us to demonstrate that PEX5 shuttles cargo completely into the peroxisomal matrix, identify features in PEX5 required for matrix entry and egress, and reveal that unfolding of PEX5 during retrieval^38^ strips off the cargo inside the organelle.^1^

The protocol below describes how to prepare *Xenopus* egg extract and use it to set up matrix protein import reactions. Import is assessed by a reporter cargo, consisting of green fluorescent protein (GFP) fused to the SKL peroxisome targeting signal (GFP-SKL). Instructions are given for depleting extract of endogenous PEX5 and adding back recombinant versions. Depletion is performed using agarose beads covalently coupled to a high-affinity PEX5-binding domain from the peroxisomal docking factor PEX14.

Before you begin, please complete the following steps first:

1. Arrange institutional permissions for housing, handling, and ovulating frogs. See institutional permissions.
2. Prime frogs before inducing ovulation. See preparation of frogs for ovulation.
3. Prepare buffers and other reagents. See materials and equipment.
4. Purify the import receptor PEX5, the fluorescent cargo GFP-SKL, and the PEX5-binding domain of PEX14. See purification of recombinant proteins.
5. Prepare beads for depleting egg extract of endogenous PEX5. See preparation of agarose beads for depleting PEX5 from egg extract.
6. Coverslips that will be used for imaging the import reactions need to be passivated to prevent protein adsorption to the glass. See preparation of passivated coverslips.

### Institutional permissions

All methods for handling *X. laevis* frogs described in this protocol have been approved by the Harvard Institutional Animal Care and Use Committee (IACUC). Readers following this protocol are advised to seek similar approval to ensure compliance with federal, state, and local government regulations and animal welfare organization guidelines.

### Preparation of frogs for ovulation

**Timing: 30 min (injection), 1 week (priming)**

Frogs should be primed with a low dose of gonadotropin to promote oocyte development. Ovulation may then be induced after several days; however, we have found that inducing ovulation after 1 week improves egg quality and yield. The benefits of priming decrease after longer durations. If institutional protocols allow, frogs should also be fasted during priming. Fasting improves egg quality by preventing defecation during egg laying, and does not adversely affect the frogs’ health within the relevant time frame.^39^

See frog priming and ovulation for a list of required materials.

**Figure 1:**
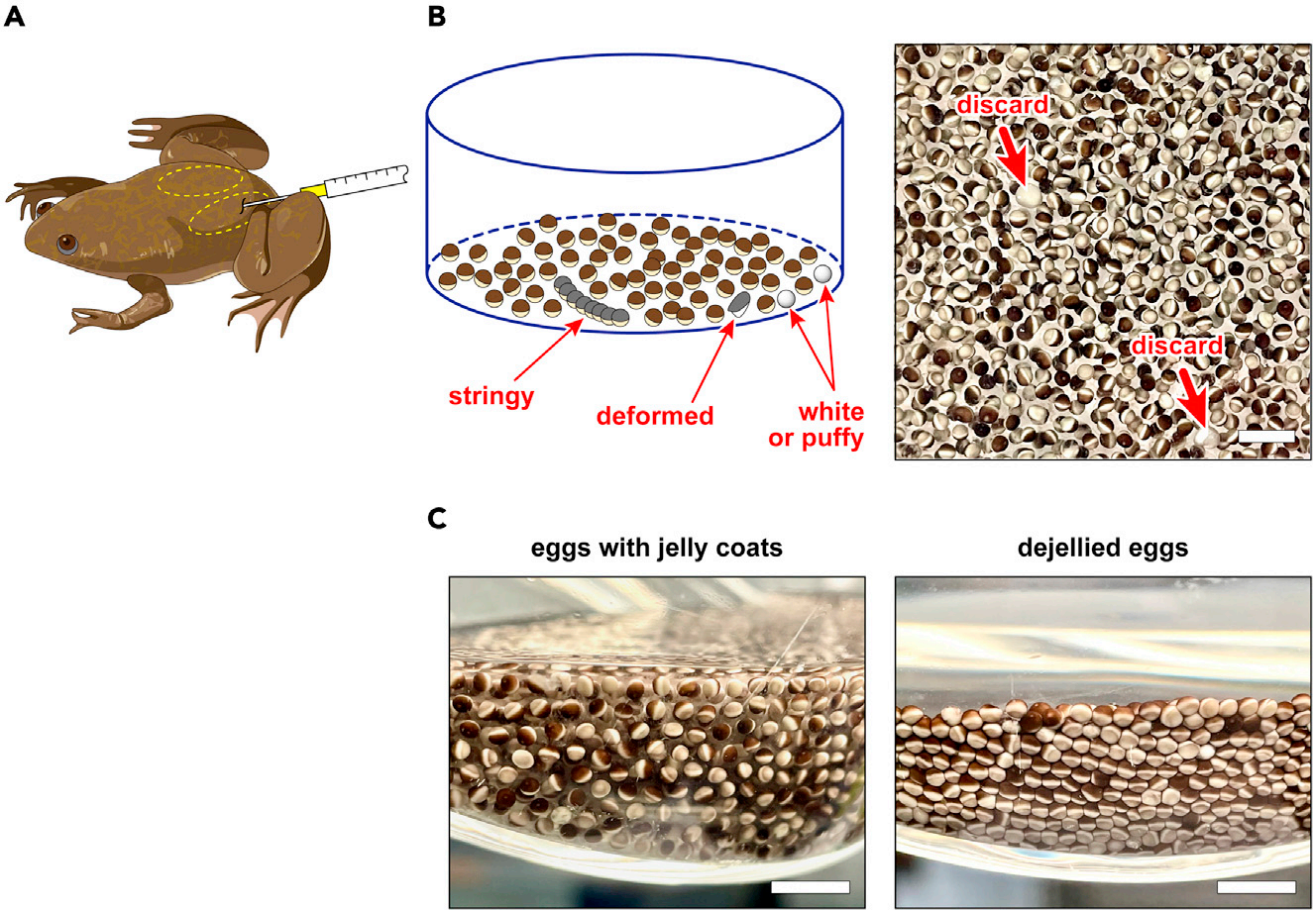
Frog injection and egg harvesting (A) Position of needle and syringe for injecting gonadotropin into the frogs’ dorsal lymph sacs, which are circled by yellow dashed lines. (B) After collecting eggs in a shallow dish, discard any that look stringy (i.e., joined together), deformed, or white and puffy. Arrows in the photograph on the right designate two puffy eggs that should be discarded. (C) How to assess dejellying efficiency. In the photograph on the left, eggs with intact jelly coats cluster loosely when the dish is tilted. In the photograph on the right, properly dejellied eggs pack tightly together. All scale bars represent 5 mm.

***Note:*** Generally, one frog will lay enough eggs to produce 1–3 mL of extract. More than one frog may be used to ensure a sufficient volume of extract for a particular experiment.

7. Inject each frog with 200 units of pregnant mare serum gonadotropin (PMSG) into the dorsal lymph sacs, using a fine 27-gauge hypodermic needle and sterile 1-mL syringe (Figure 1A).

**CRITICAL:** Discard used needles in an appropriate sharps-collection container.

8. Maintain primed frogs at 16°C in conditioned water (i.e., water purified by reverse osmosis and conditioned with rock salt approved for aquaculture).

***Note:*** At least 4 L of conditioned water are recommended per frog. Primed frogs may be housed together in a sufficiently large container.

### Purification of recombinant proteins

**Timing: 3 days (for each protein)**

Three proteins need to be purified in advance to complete this protocol: the wild-type *X. laevis* import receptor PEX5 (step 9); the fluorescent reporter cargo GFP-SKL (step 10); and the PEX5-binding domain from the *X. laevis* docking protein PEX14 (step 11). All three proteins are produced in *Escherichia coli* as N-terminal fusions to glutathione S-transferase (GST) and are purified by glutathione-affinity chromatography, as described previously.^1^ For PEX5 and GFP-SKL, the GST tag is proteolytically removed using 3C protease, generating a free N terminus with a single glycine-proline dipeptide upstream of the native initiator methionine. Removal of the tag is crucial for PEX5 because the receptor must have a free N terminus to complete the peroxisomal matrix protein import cycle.^1^ The tag is not removed from the PEX5-binding domain of PEX14 because it enhances the coupling reaction to agarose beads.

See purification of recombinant proteins for a list of required materials.

9. Purify recombinant wild-type *X. laevis* PEX5:
  a. Transform competent *E. coli* BL21 Rosetta 2(DE3) cells, according to the manufacturer’s instructions, with plasmid pMSV-013.
    ***Note:*** Select transformants on medium containing the antibiotics chloramphenicol and kanamycin. Rosetta 2 cells contain a chloramphenicol-resistant plasmid for producting tRNAs for seven rare bacterial codons. Kanamycin resistance is encoded by the protein-expression plasmid.
    ***Note:*** The expression plasmid encodes a splice variant of PEX5 called the short isoform (GenBank accession no. XP_018082765.1). This variant is fully active,^1^ but lacks a short motif needed only for import of a minor class of cargo that utilize a peroxisome targeting signal distinct from the SKL tripeptide.^21^ If desired, expression plasmids for the variant containing this motif, or for other PEX5 mutants, can be found in Skowyra and Rapoport.^1^
  b. Grow the transformed cells at 37°C in 2 L of rich medium (e.g., 2×YT) in baffled flasks, supplemented with the appropriate antibiotics, until the optical density (OD) of the culture reaches ∼0.6.
    ***Note:*** If using the recommended 2-L baffled flasks, only use 1 L of medium per flask to ensure adequate aeration of the culture.
  c. Induce expression of the recombinant protein by adding isopropyl β-D-1-thiogalactopyranoside (IPTG) to 1 mM from a 1 M stock, and incubate for an additional 16–20 h (i.e., overnight) at 16°C.
    ***Note:*** Overnight induction at low temperature reduces degradation of recombinant PEX5, which is extremely prone to proteolysis.^40^
  d. The next day, harvest the cells in 1-L bottles by centrifugation at 4,000 × *g* for 10 min at 4°C.
  e. Decant the supernatants, and resuspend the cell pellet from each liter of culture in 50 mL of cold lysis buffer.
  f. Pool the cell suspensions for a total of ∼100 mL.
  g. Supplement the pooled cell suspension with DTT to 1 mM using a 1 M stock.
    **CRITICAL:** DTT prevents oxidation of the conserved cysteine in PEX5 that is required for import.
  h. Add protease-inhibitor tablets as recommended by the manufacturer.
    **CRITICAL:** PEX5 is sensitive to proteolysis, so protease inhibitors must be included during the purification, and all subsequent steps must be performed quickly and in the cold.
  i. Lyse the cells by passing the cell suspension through a high-pressure homogenizer, such as an Avestin EmulsiFlex-C3 or a french press.
    ***Note:*** High-pressure homogenization is preferred because of the speed and efficiency of cell lysis compared to other methods.^41^ A cooling jacket, if available, helps keep the lysate cold. Alternatively, cells may be disrupted by sonication following conventional protocols.^42^
  j. Supplement the resulting cell lysate with the protease inhibitor phenylmethylsulfonyl fluoride (PMSF) to 1 mM from a 1 M stock.
    ***Note:*** PMSF is added after lysis because it is poorly soluble in aqueous solution, and undissolved PMSF crystals may clog the high-pressure homogenizer.
  k. Centrifuge the lysate at ≥ 30,000 × *g* for 30 min at 4°C to pellet cell debris and protein aggregates.
  l. Incubate the clarified lysate, with agitation, for 30 min at RT with 2–3 mL of glutathione-conjugated agarose beads equilibrated in lysis buffer.
    ***Note:*** Binding of the recombinant protein to the beads is performed at RT because of the slow binding kinetics of the GST tag to glutathione. Although binding may also be performed for 1–2 h in the cold, we have not seen significant differences in protein yield or quality after either approach.
    **CRITICAL:** Do not perform the binding step for longer than 30 min at RT.
  m. Collect the beads on a 20-mL gravity column and drain.
  n. Discard the lysate.
  o. Wash the beads with 50 mL of cold lysis buffer, then with 50 mL of cold high-salt lysis buffer, and again with 50-mL of cold lysis buffer.
    **CRITICAL:** Supplement all wash buffers with DTT to 1 mM just before use.
  p. Suspend the beads in cold lysis buffer to a final volume of 5 mL.
  q. Transfer the suspension to a 5-mL tube on ice.
  r. Supplement the suspension with DTT to 1 mM.
  s. Elute the bound protein from the beads by incubating the suspension overnight (i.e., 16–20 h) at 4°C with 1 μM of GST-tagged 3C protease.
    ***Note:*** 3C protease cleaves off the GST tag from the protein. The protease itself contains an uncleavable GST tag, which ensures that the protease will remain bound to the beads.
    **CRITICAL:** Ensure that DTT was added to the reaction. DTT prevents oxidation of the active-site cysteine in 3C protease.
  t. The next day, drain the beads in a 20-mL gravity column and collect the flow-through.
  u. Wash the beads with 4–6 mL of lysis buffer and pool the wash with the flow-through.
  v. Concentrate the pooled flow-through to ∼0.5 mL on an appropriate 10-kD centrifugal concentrator.
  w. Centrifuge the concentrated flow-through at 20,000 × *g* for 10 min at 4°C to pellet protein aggregates.
  x. Gel-filter the supernatant into XBHS buffer (supplemented with DTT to 1 mM) on a Superdex 200 10/300 GL chromatography column at 4°C.
    ***Note:*** PEX5 elutes from the column as one major peak with a retention volume of ∼11.5 mL.
  y. Pool the peak fractions and concentrate to 100 μM using an appropriate 10-kD centrifugal concentrator.
    ***Note:*** Use an extinction coefficient of 93,390 M^-1^ cm^-1^ and a molecular weight of 65 kD to calculate the protein concentration.
    ***Note:*** Expect a yield of 100–200 μL at the specified final concentration.
  z. Snap-freeze the purified protein in liquid nitrogen in single-use (e.g., 5-μL) aliquots in 0.2-mL tubes (e.g., PCR tubes), and store at −80°C.
    **CRITICAL:** Liquid nitrogen can cause severe burns. Always wear proper personal protective equipment (PPE) and follow all safety precautions when handling and transporting liquid nitrogen, as recommended by the Occupational Health and Safety Administration (OSHA).
    **CRITICAL:** Do not refreeze stock solutions of purified PEX5, because the protein is prone to degradation and aggregation after repeated freeze-thaw cycles.

10. Purify the fluorescent reporter cargo GFP-SKL:
  a. Transform competent *E. coli* BL21 Rosetta 2(DE3) cells with plasmid pMSV-064, as described above for PEX5.
    ***Note:*** The cargo is derived from a brighter and more photostable variant of monomeric enhanced GFP (mEGFP) that has been described previously.^43^
  b. Express and purify the recombinant protein, as described above for PEX5.
  c. Gel-filter the protein into XBHS buffer on a Superdex 75 10/300 GL column.
    ***Note:*** GFP-SKL elutes from the column as one major peak with a retention volume of ∼11.5 mL.
  d. Pool and concentrate the peak fractions to 100 μM using an appropriate 10-kD centrifugal concentrator.
    ***Note:*** Use an extinction coefficient of 21,890 M^-1^ cm^-1^ and a molecular weight of 27 kD to calculate the protein concentration.
    ***Note:*** Expect a yield of ∼200 μL at the specified final concentration.
  e. Snap-freeze the purified protein in liquid nitrogen in single-use (e.g., 5-μL) aliquots in 0.2-mL tubes (e.g., PCR tubes), and store at −80°C.
    **CRITICAL:** Liquid nitrogen can cause severe burns. Always wear proper personal protective equipment (PPE) and follow all safety precautions when handling and transporting liquid nitrogen, as recommended by the Occupational Health and Safety Administration (OSHA).

11. Purify the GST-tagged PEX5-binding domain of *X. laevis* PEX14:
  a. Transform competent *E. coli* BL21 Rosetta 2(DE3) cells with plasmid pMSV-099, as described above for PEX5.
    ***Note:****X. laevis* contains two redundant PEX14 genes. The PEX5-binding domain used here corresponds to amino acids 19–78 from the more highly expressed isoform (GenBank accession no. XP_018081136.1).
  b. Express the recombinant protein in 4 L of medium, as described above for PEX5.
    ***Note:*** A large quantity of the PEX5-binding domain will be needed for the coupling reaction. The purification protocol is therefore scaled up compared to the protocol for purifying PEX5 and GFP-SKL.
  c. Lyse the cells in 50 mL of cold lysis buffer per liter of culture, as described above for PEX5.
  d. Purify the recombinant protein as described above for PEX5, using 10 mL of glutathione-conjugated agarose.
    ***Note:*** Drain the beads after the binding step on a 50-mL gravity column, and use 100 mL of buffer for each wash step.
  e. Elute the GST-tagged protein by incubating the beads in 10 mL of elution buffer, supplemented with DTT to 1 mM, for 10 min at RT.
    ***Note:*** Occasionally swirl the beads to keep them suspended during the incubation.
    ***Note:*** An excess of reduced glutathione in the elution buffer must outcompete the glutathione on the beads that is bound to the GST tag. Performing the elution at RT accelerates the otherwise slow exchange kinetics of glutathione.
    **CRITICAL:** Addition of DTT prevents oxidation of glutathione.
  f. Drain the eluate into a 50-mL conical tube, and keep on ice.
  g. Repeat the elution twice more for a total of three batches.
  h. Pool all three batches of eluate for a total fo ∼30 mL.
  i. Concentrate the pooled eluate to ∼5 mL using an appropriate 10-kD centrifugal concentrator.
  j. Gel-filter the protein into XBHS buffer (supplemented with 1 mM DTT) on a prep-scale Superdex 200 HiLoad 26/600 PG chromatography column.
    ***Optional:*** If an appropriate prep-scale chromatography column is not available, the protein may be exchanged into XBHS buffer by dialysis overnight, then concentrated as described below. Use at least a 200-fold excess of buffer for the dialysis.
  k. Pool and concentrate the peak fractions to 300 μM (10 mg/mL) using an appropriate 10-kD centrifugal concentrator.
    ***Note:*** The GST-tagged protein elutes from the specified column as a dimer with a retention volume between 180 and 190 mL.
    ***Note:*** Use an extinction coefficient of 42,860 M^-1^ cm^-1^ and a molecular weight of 33 kD to calculate the protein concentration.
    ***Note:*** Expect a yield of ∼5 mL at the specified final concentration.
  l. Snap-freeze the purified protein in liquid nitrogen in single-use aliquots (e.g., 1.5-mL) in 2-mL microfuges tubes, and store at −80°C.
    **CRITICAL: Liquid nitrogen can cause severe burns.** Always wear proper personal protective equipment (PPE) and follow all safety precautions when handling and transporting liquid nitrogen, as recommended by the Occupational Health and Safety Administration (OSHA).

### Preparation of agarose beads for depleting PEX5 from egg extract

**Timing: 2 days**

To deplete endogenous PEX5 from egg extract, we use agarose beads covalently conjugated to the GST-tagged PEX5-binding domain from *X. laevis* PEX14. The beads are activated by *N*-hydroxysuccinimide esters, which react with primary amino groups in the protein to produce an amide bond. The beads are then stringently washed to remove non-covalently bound protein. As a negative control, a separate batch of beads may be coupled to GST alone using the same procedure.

See preparation of agarose beads for PEX5 depletion for a list of required materials.

12. Prepare the recombinant protein:
  a. Thaw one 1.5-mL aliquot of the purified protein (at a concentration of 10 mg/mL).
  b. Centrifuge the protein solution at 20,000 × *g* for 20 min at 4°C to pellet aggregates.
  c. Transfer the resulting supernatant to a new 2-mL microfuge tube and keep on ice.
13. Prepare the activated agarose resin:
  a. Thaw a bottle of Affi-Gel 15 activated affinity resin for 5–10 min on ice.
    ***Note:*** Adequately thawed resin should slosh around inside the bottle when swirled.
    ***Note:*** Affi-Gel 15 is preferred for coupling to proteins which have an isoelectric point (pI) less than 7, such as the PEX5-binding domain of PEX14. See the manufacturer's instructions for more information.
    **CRITICAL:** The resin is unstable in aqueous buffers due to hydrolysis of the *N*-hydroxysuccinimide ester. The resin is supplied by the manufacturer in isopropanol and should be stored at −80°C. To maximize coupling efficiency, use a fresh batch of resin, and perform all wash steps quickly using ice-cold buffers to reduce hydrolytic inactivation.
  b. Mix the resin slurry by inverting the bottle a few times.
  c. Promptly transfer 500 μL of the resin slurry to a 2-mL microfuge tube on ice, using either a wide-bore pipette tip or by directly pouring from the stock bottle.
    ***Note:*** Affi-Gel beads settle quickly by gravity. The slurry should be aliquoted soon after mixing.
    ***Note:*** Wide-bore pipette tips can be acquired commercially, or prepared when needed at the bench by cutting off a portion (∼2–3 mm) of a regular pipette tip using a clean razor blade.
    ***Note:*** The resin remaining in the bottle can be refrozen for later use. Alternatively, surplus resin may be aliquoted and frozen in smaller volumes (e.g., 500 μL) to avoid multiple freeze-thaw cycles.
  d. Let the resin settle on ice, then adjust the bed volume to 500 μL.
  e. Aspirate off the isopropanol.
  f. Add 1 mL of ice-cold MilliQ-grade water over the resin to resuspend, then invert the tube a few times to mix.
  g. Collect the resin by centrifugation at 1,500 × *g* for 1 min at 4°C, and immediately place on ice.
    ***Note:*** A swinging bucket rotor (e.g., Eppendorf model S-24-11-AT) facilitates resin collection.
  h. Wash the resin once more with ice-cold water.
  i. Wash the resin 3 times with 1 mL of ice-cold XBH buffer.
14. Set up the coupling reaction:
  a. After the last wash, completely aspirate off the supernatant.
    ***Note:*** Aspirating off the supernatant is facilitated by using a flattened capillary pipette tip, which can be prepared by pinching 1-2 mm of the end of the tip with a pair of clean pliers.
  b. Immediately add 1.5 mL of the cold purified protein (10 mg/mL) over the resin.
  c. Invert the tube a few times to mix.
  d. Rotate the tube in the cold room overnight (i.e., 16–20 h).
    ***Note:*** The specified amounts yield 30 mg of protein per milliter of resin, at a 3:1 volume ratio of protein solution to resin. These proportions are recommended by the manufacturer for optimal coupling efficiency.

15. Wash off unbound protein:
  a. Pour the resin into a clean Poly-Prep 10-mL gravity column and drain.
  b. Discard the flow-through.
  c. Wash the resin with 10 mL of cold XBH buffer, then with 10 mL of cold XBH buffer (high-salt), and finally with 10 mL of additional cold XBH buffer.
  d. Wash the resin with 4 mL of cold glycine buffer (pH 2.4), then immediately with 10 mL of XBH buffer to neutralize the pH.
  e. Repeat the glycine wash twice more.
  f. Ensure the absence of protein in the final flow-through using a Bradford assay.
  g. Plug the column and resuspend the resin in an equal volume of XBHS.
  h. Transfer the resulting 1:1 suspension to a 1.5-mL microfuge tube, and store at 4°C.

***Note:*** The coupled resin can be stored at 4°C for several months.

### Preparation of passivated coverslips

**Timing: 2 days**

Passivating coverslips with polyethylene glycol (PEG) creates a hydrophilic surface that minimizes adsorption of extract components to the glass during imaging. Bonding with the glass occurs through an activated methoxy silane functional group on the PEG. To ensure adequate bonding, coverslips are thoroughly cleaned of residual organic material. We prefer oxygen plasma for this purpose because the procedure is quick and efficient. Suitable alternatives are described in Field et al.^37^

See passivating coverslips for a list of required materials.

16. Clean the coverslips:
  a. Arrange the coverslips in a coverslip rack.
  b. Rinse the coverslips with MilliQ-grade water to remove dust and salts.
    ***Note:*** To immobilize the coverslips in the rack during washing, push along one edge of the coverslips with the index finger to press them against the rack (Figure 2A).
    **Figure 2:**
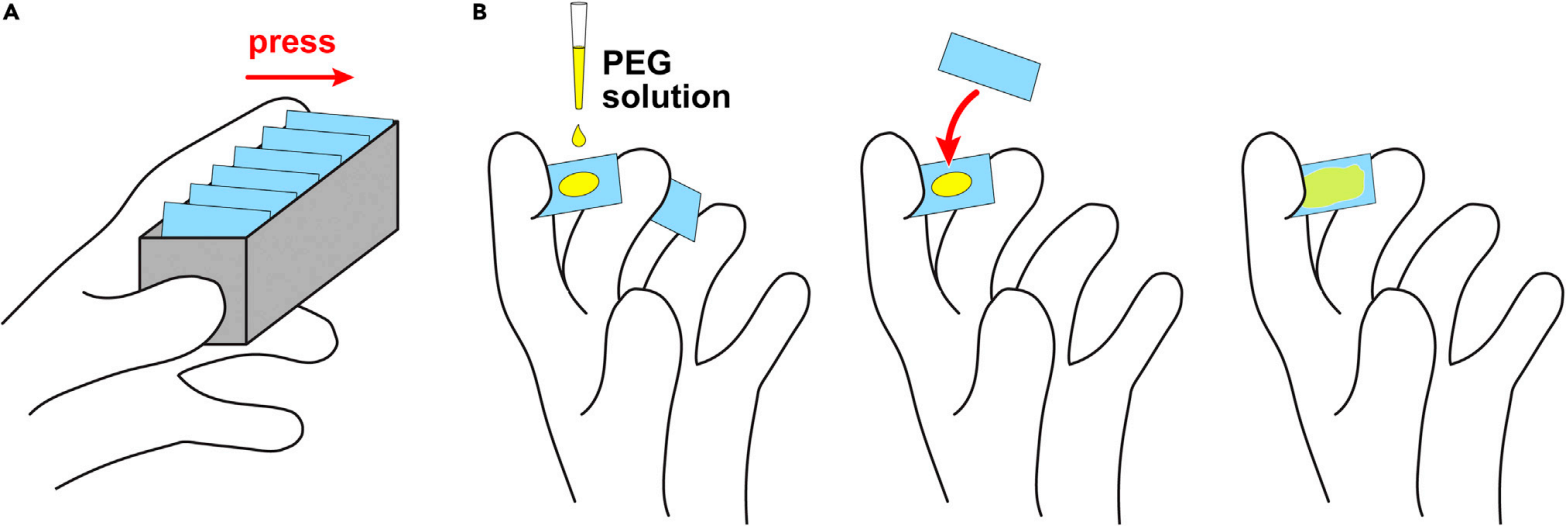
Preparation of passivated coverslips (A) Immobilizing coverslips in a coverslip rack by pressing along one edge with the index finger. (B) Preparation of PEGylated coverslip sandwiches. Two clean coverslips are held between the fingers of one hand as shown. A solution of PEG is dropped onto one of the coverslips with the other hand, and the second coverslip is immediately placed over the drop to form a sandwich.
  c. Blow off excess water with compressed air or a handheld rubber bellows.
  d. Dry in a hot oven or on top of a hot plate.
  e. Expose the washed and dried coverslips to oxygen plasma for 4 min at 200 W and a partial oxygen pressure of 0.1 Torr, and proceed immediately to bonding.
    ***Note:*** These parameters have been optimized for the 500 II plasma etcher (from Technics). Optimal parameters on other devices should be determined empirically.

17. Bond the cleaned coverslips to PEG:
  a. Warm up the methoxy PEG silane to RT.
    ***Note:*** This step can be done in advance, e.g., while the coverslips are being cleaned.
  b. Weigh out 30–40 mg of methoxy PEG silane into a 1.5-mL microfuge tube.
  c. Add 900 μL of the PEG solvent.
  d. Heat the tube for a few seconds in a block heater warmed to 80°C.
  e. Once the PEG dissolves, mix the tube a few times by inversion.
  f. Open the tube and keep at 80°C.
  g. Pipette 20 μL of the PEG solution onto the center of one coverslip, then promptly place another coverslip on top to form a sandwich.
    ***Note:*** Ensure that the PEG solution has spread evenly over the inner surface of the sandwich. The presence of small bubbles is fine as they will rise up during the subsequent incubation.
    ***Note:*** This step should be performed quickly to prevent the PEG solution from cooling on the coverslip. Cooled PEG solution might not spread evenly over the glass. A recommended procedure is illustrated in Figure 2B: two coverslips are held in one hand, the PEG solution is pipetted onto one of them, and the other coverslip is immediately placed on top. Alternatively, the sandwich may be assembled by removing a fresh coverslip from the rack and placing it on top of the first, although care should be taken to work promptly in this case.
  h. Place the assembled sandwich upright in a coverslip rack.
  i. Repeat with the remaining coverslips until the rack is full.
  j. Place the rack in an oven pre-warmed to 80°C and incubate overnight (i.e., 16–20 h).
  k. The following day, remove the racks from the oven and store at RT in a closed container (e.g., an empty pipette-tip box).
    ***Note:*** The prepared coverslips are usable for at least 1 month.

## Key resources table

**Table:** 

REAGENT or RESOURCE	SOURCE	IDENTIFIER
**Bacterial and virus strains**		

*E. coli* BL21 Rosetta 2(DE3)	Millipore-Sigma	Cat. # 71397

**Chemicals, peptides, and recombinant proteins**		

2×YT medium (granulated)	Thermo Fisher	Cat. # BP9743
Acetic acid (glacial)	Fisher	Cat. # A491-212
CaCl_2_·2H_2_O (calcium chloride dihydrate)	Millipore-Sigma	Cat. # C3306
Chloramphenicol	Millipore-Sigma	Cat. # C0378
Chymostatin	Millipore-Sigma	Cat. # C7268
Cytochalasin D	Millipore-Sigma	Cat. # C8273
Cycloheximide	Millipore-Sigma	Cat. # C7698
Cysteine	Millipore-Sigma	Cat. # W326305
DMSO (dimethylsulfoxide), anhydrous	Millipore-Sigma	Cat. # 900645-4X2ML
DTT (dithiothreitol)	GoldBio	Cat. # DTT
EDTA (ethylenediaminetetraacetic acid)	Millipore-Sigma	Cat. # E9884
Ethanol (absolute)	Koptec	Cat. # V1016
Glutathione (reduced)	Millipore-Sigma	Cat. # G4251
Glycine	Fisher	Cat. # BP3815
GST-tagged 3C protease	homemade^44^	pGEX-4T-1/HRV
HCl (hydrochloric acid)	Fisher	Cat. # A144-212
HEPES (free acid)	Millipore-Sigma	Cat. # H3375
Human chorionic gonadotropin (HCG)	Merck	Cat. # 133754
IPTG (isopropyl β-D-1-thiogalactopyranoside)	GoldBio	Cat. # I2481C
Kanamycin	Millipore-Sigma	Cat. # K4000
KCl (potassium chloride)	Millipore-Sigma	Cat. # P9541
KOH (potassium hydroxide)	Millipore-Sigma	Cat. # P5958
Lanolin	Millipore-Sigma	Cat. # L7387
Leupeptin hemisulfate	Millipore-Sigma	Cat. # L2884
Methoxy PEG silane (20-kD)	JenKem	Cat. # M-SLN-20K
MgCl_2_·6H_2_O (magnesium chloride hexahydrate)	Millipore-Sigma	Cat. # M2670
NaCl (sodium chloride)	Millipore-Sigma	Cat. # S7653
NaOH (sodium hydroxide)	Millipore-Sigma	Cat. # 221465
Paraffin wax	Millipore-Sigma	Cat # 327204
PBS tablets (phosphate-buffered saline)	Millipore-Sigma	Cat. # P4417
Pepstatin A	Millipore-Sigma	Cat. # P5318
PMSF (phenylmethylsulfonyl fluoride)	Millipore-Sigma	Cat. # P7626
Pregnant mare serum gonadotropin (PMSG)	BioVendor	Cat. # RP1782725000
Protease inhibitor tablets	Millipore-Sigma	Cat. # 11873580001
Sucrose	Millipore-Sigma	Cat. # S7903
Tris (free base)	Millipore-Sigma	Cat. # 93362
Vaseline	Millipore-Sigma	Cat. # 16415

**Critical commercial assays**		

Affi-Gel 15 activated agarose resin	Bio-Rad	Cat. # 1536051
Bradford 1× dye reagent	BioRad	Cat. # 5000205
Glutathione-conjugated agarose (Sepharose 4B)	Cytiva	Cat. # 17075605
Superdex 200 Increase 10/300 GL column	Cytiva	Cat. # 28990944
Superdex 200 HiLoad 26/600 PG column	Cytiva	Cat. # 28989336
Superdex 75 Increase 10/300 GL column	Cytiva	Cat. # 29148721

**Experimental models: Organisms/strains**		

*Xenopus laevis* African wild-type, pigmented, adult female clawed frogs	Amphibian/Aquatics facility at Harvard Medical School	N/A

**Recombinant DNA**		

Plasmid for bacterial expression of *X. laevis* PEX5	Skowyra and Rapoport^1^	pMSV-013
Plasmid for bacterial expression of GFP-SKL	Skowyra and Rapoport^1^	pMSV-064
Plasmid for bacterial expression of the PEX5-binding domain from *X. laevis* PEX14	Skowyra and Rapoport^1^	pMSV-099

**Software and algorithms**		

ImageJ (Fiji distribution)	Schneider et al.^45^	https://imagej.net/software/fiji/

**Other**		

1-L centrifuge bottles (polypropylene) with caps	Beckman Coulter	C31597
2-L baffled flasks	Bellco Glass	Cat. # 2542-02000
0.2-mL PCR tubes	USA Scientific	Cat. # 1402-8100
0.5-mL microfuge tubes	USA Scientific	Cat. # 1405-8100
1.5-mL microfuge tubes	USA Scientific	Cat. # 1615-5500
2-mL microfuge tubes	USA Scientific	Cat. # 1420-2700
5-mL microfuge tubes	USA Scientific	Cat. # 4011-8302
1-mL syringe (all-plastic, luer tip, sterile)	Henke-Sass-Wolf	Cat. # 4010-200V0
18g needle (1.5-inch, beveled tip, sterile)	Becton Dickinson	Cat. # 305196
27g hypodermic needle (0.5-inch, beveled tip, sterile)	Covidien	Cat. # 8881250362
Block heater (with metal blocks for 1.5-mL tubes)	Cole-Parmer	Cat. # SK-36620-09
Centrifugal concentrators	Amicon	
Centrifuge (refrigerated), model 5427 R	Eppendorf	Cat. # 5409000136
Centrifuge rotor (swinging-bucket), model S-24-11-AT	Eppendorf	Cat. # 5409715003
Centrifuge (touchspin)	Southwest Science	Cat. # SC1006-M
Cotton swabs	Puritan	Cat. # 868WCS
Coverslips (22 × 22 mm, square, # 1.5 glass)	VWR	Cat. # 48366-227
Coverslip rack	EMS Diasum	Cat. # 72240
Dish (glass)	VWR	Cat. # 89000-294
EmulsiFlex-C3 high-pressure homogenizer	Avestin	
FPLC system (ÄKTA)	Cytiva	
Gravity column, 20-mL (15 × 100 mm, glass)	Kimble Chase	Cat. # 420400-1510
Gravity column, 50-mL (25 × 100 mm, glass)	Kimble Chase	Cat. # 420400-0730
Hot plate	Corning	Cat. # 6797-400D
Incubator oven (gravity)	Thermo Fisher	Cat. # 151030520
Micro-Spin columns (800-μL)	Pierce	Cat. # 89868
Nanodrop microvolume spectrophotometer	Thermo Fisher	
Plasma etcher	Technics	Model # 500 II
Poly-Prep gravity columns	Bio-Rad	Cat. # 731-1550
Razor blades (single-edge)	Techniedge	Cat. # TE05-091C
Rotator	Thermo Scientific	Cat. # 11-676-341SD
Rubber bellows (hand-held)	Giottos	Model # AA1910
Slides (aluminum)	homemade	See Figure 5
Slide tray (with lid)	Thermo Fisher	Cat. # 12-587-10
Spinning-disk confocal fluorescence microscope	N/A	N/A
Thermomixer	Eppendorf	Cat. # 5382000023
Thermoblock (96-well), for thermomixer	Eppendorf	Cat. # 5306000006
Transfer pipets, 2-mL (soft polyethylene)	Thermo Fisher	Cat. # 13-711-9AM
Tweezers (fine-tipped, style 55)	Dumont	Cat. # 0209-55-PO
Ultracentrifuge	Beckman Coulter	
Ultracentrifuge rotor (swinging-bucket), model SW 55 Ti	Beckman Coulter	Cat. # 342194
Ultracentrifuge tubes, 5-mL (13 × 51 mm, thin-wall ultraclear polycarbonate)	Beckman Coulter	Cat. # 344057

## Materials and equipment

Prepare all solutions in MilliQ-grade water from high-purity reagents. RT denotes room temperature (23°C–25ºC).

### Buffer stocks

**Table:** MMR buffer (20×)

Reagent	Final concentration			
	20×	1×	Amount for 1 L	
HEPES	100 mM	5 mM	23.83 g	free acid
MgCl_2_·6H_2_O	20 mM	1 mM	4.065 g	
CaCl_2_·2H_2_O	40 mM	2 mM	5.88 g	
KCl	40 mM	2 mM	2.98 g	
NaCl	2 M	100 mM	116.88 g	
EDTA	2 mM	0.1 mM	0.585 g	free acid
Water	N/A	N/A	up to 1 L	

Adjust the pH to 7.8 at RT with NaOH. Sterile filter and store for up to 1 year at RT.

**Table:** XB buffer (20×)

Reagent	Final concentration			
	20×	1×	Amount for 1 L	
HEPES	200 mM	10 mM	47.66 g	free acid
MgCl_2_·6H_2_O	20 mM	1 mM	4.065 g	
CaCl_2_·2H_2_O	2 mM	0.1 mM	0.294 g	
KCl	2 M	100 mM	149.1 g	
Water	N/A	N/A	up to 1 L	

Adjust the pH to 7.8 at RT with KOH. Sterile filter and store for up to 1 year at RT.

#### EDTA (0.5 M, pH 8)

Dissolve 73.06 g of the free-acid form of EDTA in 400 mL of water. Titrate the EDTA into solution by slowly raising the pH to ∼7.5 with NaOH pellets. Adjust the final pH to 8.0 at RT with NaOH. Bring the volume up to 500 mL, sterile filter, and store for up to 1 year at RT.

#### HEPES buffer (1 M, pH 7.8)

Dissolve 238.3 g of the free-acid form of HEPES in 700 mL of water. Adjust the pH to 7.8 at RT with KOH. Bring the volume up to 1 L, sterile filter, and store for up to 1 year at RT.

#### Tris buffer (1 M, pH 7.5)

Dissolve 121.14 g of the free-base form of Tris in 700 mL of water. Adjust the pH to 7.5 at RT (8.0 at 4°C) with HCl. Bring the volume up to 1 L, sterile filter, and store for up to 1 year at RT.

#### Sucrose (2 M)

Dissolve 684.6 g of sucrose in water up to 1 L. Complete dissolution of the sucrose might take 1-2 h at RT, and may be accelerated by mild heating. Sterile filter and store for up to 1 year at RT. If the sucrose solution is too dense to pass through a 0.2-μm filter, a 0.4-μm filter may be used. In this case, store the final solution at 4°C to prevent microbial growth.

#### KCl (2 M)

Dissolve 149.1 g of KCl in water up to 1 L. Sterile filter and store for up to 1 year at RT.

#### NaCl (2 M)

Dissolve 116.88 g of NaCl in water up to 1 L. Sterile filter and store for up to 1 year at RT.

### Frog priming and ovulation

#### Hormone stocks

Dissolve pregnant mare serum gonadotropin (PMSG) to 400 units / mL in sterile 1× PBS, and store frozen in single-use (e.g., 500-μL) aliquots. Do not refreeze PMSG solutions, and discard any unused stocks after the expiration date specified by the manufacturer.

Dissolve human chorionic gonadotropin (HCG) to 1,000 units / mL in sterile 1× PBS, and store frozen in single-use (e.g., 500-μL) aliquots. Do not refreeze HCG solutions, and discard any unused stocks after the expiration date specified by the manufacturer.

#### PBS (1×)

Dissolve 5 PBS tablets in 1 L of water, sterile filter, and store for up to 1 year at RT.

#### MMR (1×)

For 1 L, dilute 50 mL of 20× MMR stock (see above) in 950 mL of water pre-equilibrated to 16°C–18ºC (to avoid stressing the frogs and eggs). Prepare a sufficient volume just before use for the total number of frogs used for ovulation. Do not store this solution to avoid microbial growth that could be harmful to the frogs.

### Purification of recombinant proteins

**Table:** Lysis buffer

Reagent	Final concentration	Amount for 1 L	
Tris	50 mM	50 mL	1 M stock, pH 7.5 (see above)
NaCl	150 mM	75 mL	2 M stock (see above)
EDTA	1 mM	2 mL	0.5 M stock (see above)
water	N/A	873 mL	

Adjust the pH to 7.5 at RT (pH 8.0 at 4°C) with NaOH, then chill at 4°C before use. The buffer may be stored for up to 1 year.

**Table:** Lysis buffer (high-salt)

Reagent	Final concentration	Amount for 1 L	
Tris	50 mM	50 mL	1 M stock, pH 7.5 (see above)
NaCl	600 mM	300 mL	2 M stock (see above)
EDTA	1 mM	2 mL	0.5 M stock (see above)
water	N/A	648 mL	

Adjust the pH to 7.5 at RT (pH 8.0 at 4°C) with NaOH, then chill at 4°C before use. The buffer may be stored for up to 1 year.

**Table:** Elution buffer

Reagent	Final concentration	Amount for 40 mL	
Tris	50 mM	2 mL	1 M stock, pH 7.5 (see above)
NaCl	150 mM	3 mL	2 M stock (see above)
EDTA	1 mM	80 μL	0.5 M stock (see above)
glutathione	40 mM	490 mg	reduced form
water	N/A	up to 40 mL	

Adjust the pH to 7.5 at RT with NaOH. There is no need to chill this buffer. Prepare just before use, and do not store this buffer because glutathione may oxidize over time.

**Table:** XBHS buffer

Reagent	Final concentration	Amount for 1 L	
XB buffer	1×	50 mL	20× stock (see above)
HEPES	30 mM	30 mL	1 M stock, pH 7.8 (see above)
sucrose	250 mM	125 mL	2 M stock (see above)
water	N/A	up to 1 L	

Adjust the pH to 7.8 at RT with KOH, then sterile filter and store for up to 1 year at 4°C. Note that XB buffer at 1× already contains 10 mM HEPES. We supplement XBHS buffer with 30 mM additional HEPES to reach a total concentration of 40 mM.

#### DTT (1 M)

Dissolve 3.08 g of DTT in water up to 20 mL. Store frozen for up to 1 year in single-use aliquots (e.g., 1 mL).

#### IPTG (1 M)

Dissolve 0.24 g of IPTG in water up 1 mL just before use. Do not store this solution.

#### PMSF (100 mM)

Dissolve 17.4 mg of PMSF in 1 mL of absolute (100% v/v) ethanol just before use. Do not store this solution.

### Preparation of agarose beads for PEX5 depletion

#### XBH buffer

1× XB buffer supplemented with HEPESKOH (pH 7.8 at RT) to 30 mM. For 1 L, mix 50 mL of 20× XB buffer stock (see above) and 30 mL of 1 M HEPES buffer (pH 7.8) stock (see above) in 900 mL of water. Adjust the pH to 7.8 at RT with KOH, bring the volume up to 1 L, then sterile filter and store for up to 1 year at 4°C. Note that XB buffer at 1× already contains 10 mM HEPES; we supplement XBH buffer with 30 mM additional HEPES to reach a total concentration of 40 mM.

**Table:** XBH buffer (high-salt)

Reagent	Final concentration	Amount for 1 L	
XB buffer	1×	50 mL	20× stock (see above)
HEPES	30 mM	30 mL	1 M stock, pH 7.8 (see above)
KCl	1 M	450 mL	2 M stock (see above)
water	N/A	up to 1 L	

Adjust the pH to 7.8 at RT with KOH, then sterile filter and store for up to 1 year at 4°C. Note that XB buffer at 1× already contains 10 mM HEPES and 100 mM KCl. We supplement high-salt XBH buffer with 30 mM additional HEPES and 900 mM additional KCl to reach each respective final concentration.

#### XBHS buffer

See above.

#### Glycine buffer (pH 2.4)

100 mM glycine and 150 mM KCl. For 1 L, dissolve 7.5 g of glycine in 800 mL of water. Add 75 mL of 2 M KCl stock (see above), and adjust the pH to 2.4 at RT with HCl. Bring the volume up to 1 L, sterile filter, and store for up to 1 year at 4°C.

### Passivating coverslips

#### PEG solvent

95% v/v ethanol, 5% v/v water, and 0.1% v/v acetic acid. Add 47.5 mL of absolute (100% v/v) ethanol, 2.5 mL of water, and 50 μL of glacial acetic acid, in the specified order, to a 50-mL conical tube. Close the tube and mix well by inversion. Store tightly capped (to prevent evaporation) for up to 1 year at RT.

### Preparation of egg extract

**Table:** Dejellying solution

Reagent	Final concentration	Amount for 1 L	
MMR buffer	1×	50 mL	20× stock (see above)
sucrose	50 mM	25 mL	2 M stock (see above)
cysteine	20 g/L	20 g	
NaOH	25 mM	1 g	
water	N/A	up to 1 L	

Verify that the pH is between 7.5 and 8. Prepare just before use to avoid oxidation of the cysteine. Do not store this solution.

#### XBS buffer

1× XB buffer supplemented with sucrose to 50 mM. For 1 L, mix 50 mL of 20× XB buffer stock (see above) and 25 mL of 2 M sucrose stock (see above) in 925 mL of water. Prepare just before use. Do not store this buffer.

**Table:** Protease inhibitor cocktail with cytochalasin and cycloheximide (1000× stock)

Reagent	Final concentration			
	1000×	1×	Amount for 500 μL	
chymostatin	10 mg/mL	10 μg/mL	5 mg	
leupeptin	10 mg/mL	10 μg/mL	5 mg	
pepstatin A	10 mg/mL	10 μg/mL	5 mg	
cytochalasin D	10 mg/mL	10 μg/mL	5 mg	
cycloheximide	100 mg/mL	100 μg/mL	50 mg	
DMSO	N/A	N/A	up to 500 μL	anhydrous

Store frozen for up to 1 year in single-use (e.g., 10-μL) aliquots.

### Depletion of PEX5

#### XBH buffer

See above.

### Setting up an import reaction

#### XBHS buffer

See above.

### Imaging the import reactions

#### Valap sealant

33.3% w/v each of vaseline, lanolin, and paraffin wax. For ∼300 mL, Weigh 100 g each of vaseline, lanolin, and paraffin wax into a 1-L glass beaker. The reagents can be scooped out from the stock containers with a spatula. Cover the beaker with aluminum foil and heat at 65°C–70ºC on a hot plate for 1–2 h until all reagents have melted. Mix occasionally by swirling to avoid burning. The final solution should have a golden-yellow color, and may be poured into smaller containers for storage. Store for up to 1 year at RT. Before using, reheat at ≥ 70°C until melted.

## Step-by-step method details

### Inducing ovulation

**Timing: 2 h**

Egg extract is prepared from freshly harvested eggs on the day of the experiment. To trigger egg laying, primed frogs must be injected the day before with a high dose of gonadotropin.

See frog priming and ovulation for a list of required materials.

1. One day before the experiment, induce ovulation by injecting 500 units of human chorionic gonadotropin (HCG) into the dorsal lymph sacs of each primed frog, using a fine 27-gauge hypodermic needle and sterile 1-mL syringe (Figure 1A).

***Note:*** To improve egg yield, frogs should be injected between 16–24 h before egg extract preparation.

**CRITICAL:** Discard used needles in an appropriate sharps-collection container.

2. Transfer each frog into 2 L of 1× MMR buffer and house overnight at 16°C in separate containers.

***Note:*** MMR buffer^46^ improves the quality of the eggs and helps prevent premature activation of the eggs before they can be collected.^47^ Please see the next section on preparation of egg extract for more information.

**CRITICAL:** Frogs should not be maintained in MMR buffer for longer than 16 h to avoid osmotic stress. If a longer ovulation period is desired, frogs should be injected earlier in the day and maintained in conditioned water before being transferred into MMR later in the day.

### Preparation of egg extract

**Timing: 2 h**

Egg extract corresponds to frog egg cytoplasm, and is generated by quickly crushing the eggs by centrifugation. It is imperative to lock the extract in interphase of the cell cycle for the import reactions to work, because peroxisomal matrix protein import is turned off during cell division by mitotic cyclin-dependent kinases.^48^ Freshly harvested eggs are arrested in metaphase,^49^ but progress into interphase upon being crushed because of the influx of calcium from residual buffer used for harvesting. Extract can be locked in interphase by the addition of cycloheximide,^37^ which prevents the synthesis of cyclins needed to enter the next cell cycle.

See preparation of egg extract for a list of required materials.

3. The following morning, transfer the frogs to a new container filled with at least 4 L of conditioned water per frog.

***Note:*** Frogs may be housed together in a sufficiently large container. The frogs should complete ovulation for an additional 24 h at 16°C, and any additional eggs laid during this time should be discarded. The frogs should then be allowed to recover for 3–4 months, according to institutional protocols, before being reused for ovulation.

**CRITICAL:** The recovery period is essential to avoid harming the frogs' reproductive system.

4. Carefully decant the eggs into a round glass dish, beaker, or other suitable container.
5. Examine the eggs, and use a 2-mL transfer pipet to remove any that look malformed or damaged.

***Note:*** Healthy eggs look spherical, are enclosed in a transparent jelly coat, and have one hemisphere colored white (the vegetal pole) and the other dark (the animal pole). Eggs that look uniformly white and swollen (i.e., puffy) should be discarded, as should eggs that are joined together (stringy) or are otherwise deformed or discolored (Figure 1B).

**Figure 3:**
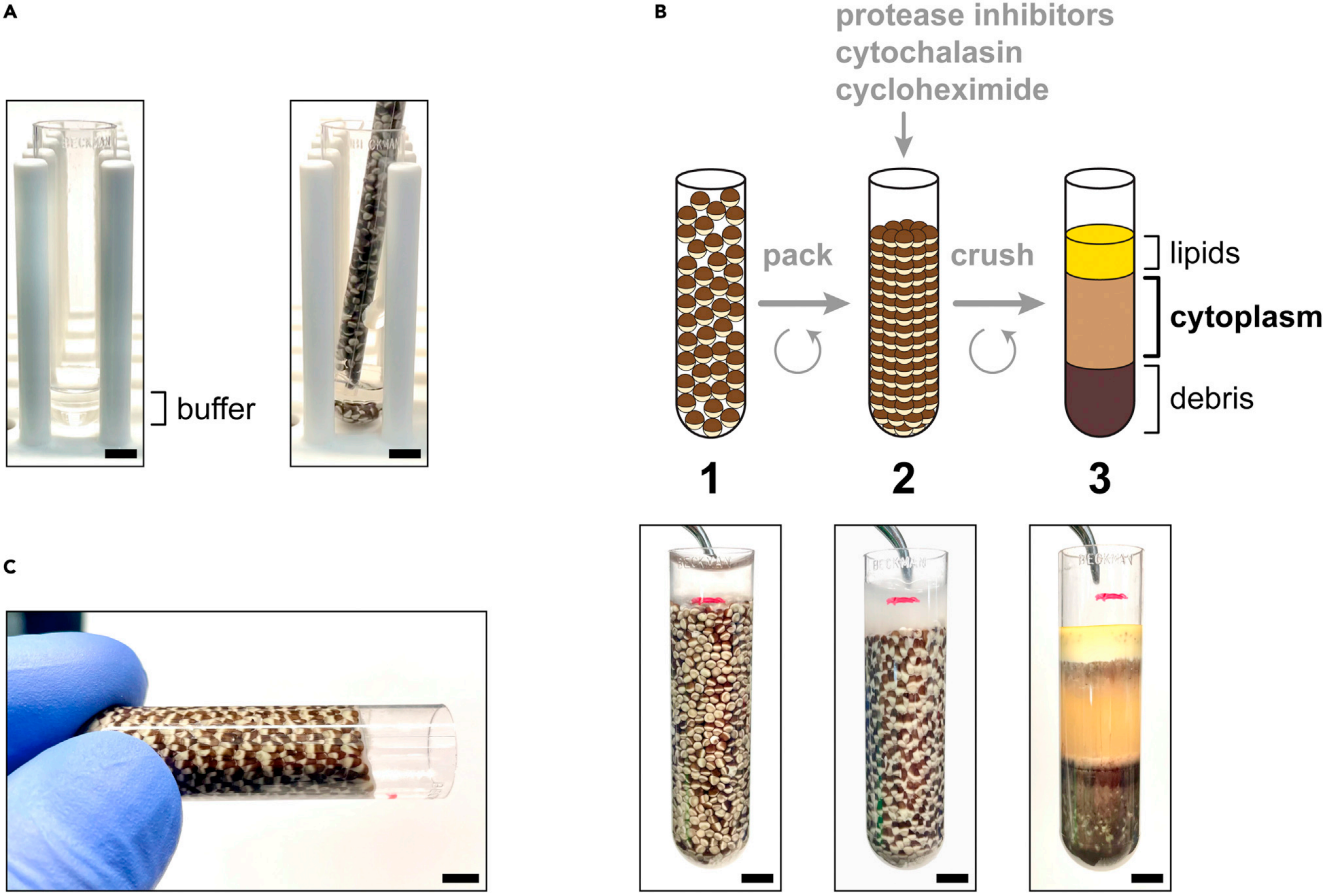
Fractionating eggs to produce a cytoplasmic extract (A) Transferring eggs to a thin-wall tube mounted in a test tube rack. A little buffer (∼1 mL) is first added to the tube, then eggs are dispensed from a transfer pipet. Note how the pipette is immersed in the buffer before expelling the eggs. (B) Scheme on top depicts the procedure for fractionating eggs. Step 1, eggs are transferred to a thin-wall tube as described above. Step 2, eggs are packed by low-speed centrifugation, then supplemented as shown. Step 3, packed eggs are crushed by high-speed centrifugation to produce a lipid layer on top, a straw-colored cytoplasmic fraction (i.e., the extract) in the middle, and a dark debris pellet on the bottom. Photographs show the actual appearance of the eggs after each step. (C) Tilt test to assess egg compaction. Properly packed eggs resist sliding when the tube is tilted. All scale bars represent 5 mm.
  b. Cut off the tip of a 2-mL soft polypropylene transfer pipet.
  c. Tilt the dish and collect the eggs along one side.
  d. Carefully scoop up some eggs using the prepared pipet and deposit in the tube.
    ***Note:*** To avoid disrupting the eggs by surface tension, immerse the transfer pipet in the buffer already in the bottom of the tube and dispense the eggs into the buffer (Figure 3A, right image).
  e. Repeat until the tube is almost completely full with eggs (Figure 3B, step 1).
    ***Note:*** Multiple tubes may be used to process more eggs. However, tubes should not be filled less than half-way to prevent collapse during centrifugation. If more tubes are necessary, the volume of eggs should be adjusted (by eye) to be similar in each tube.
  f. Aspirate off most of the buffer covering the eggs.
  g. Centrifuge the tube at 16°C, first at 350 × *g* for 1 min, then at 1,200 × *g* for an additional minute (Figure 3B, step 2).
    ***Note:*** Packing the eggs displaces most of the buffer that could otherwise dilute the final egg extract. Properly packed eggs should be tightly squeezed against each other but remain otherwise intact (Figure 3B, step 2). They should also be fixed in place and not slide around when the tube is tilted (Figure 3C).
    ***Note:*** Performing this step in two stages ensures optimal packing efficiency: the initial low-speed spin settles the eggs in the tube, while the second higher-speed spin compresses the eggs to fill most of the interstitial space. We start the centrifuge at 350 × *g* set for 2 min; after the first minute, we quickly increase the speed to 1,200 × *g* and let the run complete in the remaining time.

**Figure 4:**
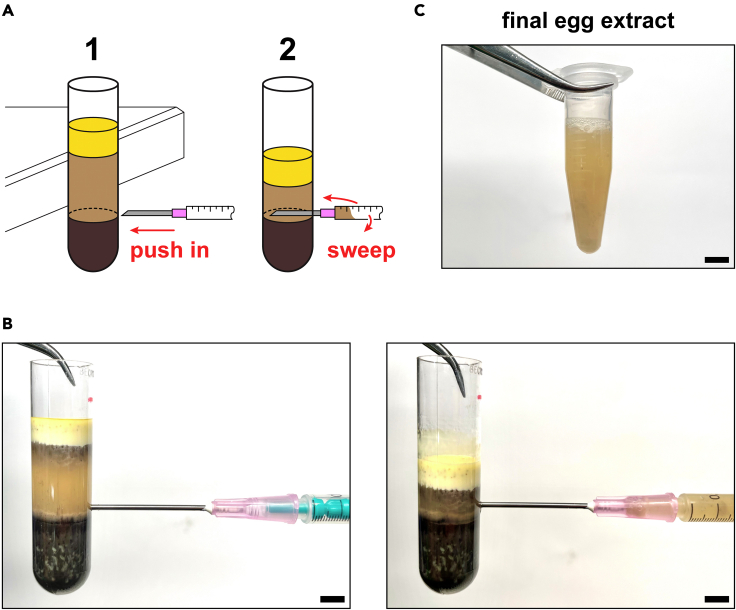
Removal of the cytoplasmic extract after fractionating eggs (A) Scheme depicting the procedure for isolating the cytoplasmic fraction (i.e., the extract) after fractionating eggs. Step 1, the tube is propped against the edge of the bench as shown, then pierced by a large-gauge needle just above the dark pellet on the bottom. Step 2, the cytoplasmic fraction is aspirated out while sweeping the needle from side to side. (B) Photographs illustrating a pierced tube before (left) and after (right) aspirating out the cytoplasmic fraction. (C) The resulting egg extract in a microfuge tube. All scale bars represent 5 mm.

6. Dejelly the eggs:
  a. Decant most of the buffer from the glass dish, leaving some to cover the eggs.
  b. Pour in a third of the dejellying solution, and gently swirl the dish for 1–2 min.
    ***Note:*** Adding the dejellying solution in portions gradually changes the buffer composition and thereby reduces stress on the eggs.
    ***Note:*** Inclusion of cysteine (a mild reductant) in the dejellying solution helps disrupt the disulfide bonds between mucin molecules that hold the jelly coats together.^50^
  c. Decant most of the buffer, and repeat with the next third of dejellying solution.
  d. Decant most of the buffer, and add the last third of dellying solution.
  e. Swirl the dish for 10–15 min, or until the eggs have shed their jelly coats.
    ***Note:*** The transparent jelly coats are difficult to see, but appear as a slightly refractive layer encasing the eggs. A better method for judging dejellying efficiency is to tilt the dish so that the eggs pile up on one side (Figure 1C). Dejellied eggs pack tightly against each other without noticeable interstitial space.
    **CRITICAL:** Dejellying should be complete within 10–15 min. If this step takes significantly longer (> 25–30 min), the prolonged exposure of the eggs to cysteine can compromise the quality of the extract. See problem 1 in the troubleshooting section for more information.
  f. Decant most of the buffer.
  g. Pour in 200 mL of XBS buffer and swirl for 2 min.
    ***Note:*** Adding XBS buffer in portions gradually changes the buffer composition and thereby reduces stress on the eggs.
  h. Decant most of the buffer, and repeat three more times with additional XBS buffer.
  i. Pour in the last 200 mL of XBS buffer.

7. Pack the eggs:
  a. Add 1 mL of XBS buffer to a 5-mL thin-walled ultracentrifuge tube (Figure 3A, left image).

8. Crush the eggs:
  a. Aspirate off as much residual buffer as possible, taking care not to disrupt the packed eggs.
  b. Estimate the volume of packed eggs, and add an appropriate volume of the 1000× stock solution of protease inhibitors, cytochalasin, and cycloheximide.
    ***Note:*** The stock can be directly pipetted onto the top of the packed eggs (Figure 3B, step 2).
    ***Note:*** Cytochalasin prevents actin polymerization and thereby decreases the viscosity of the extract to facilitate pipetting. Cycloheximide locks the extract in interphase, and is crucial for the import reactions to work because peroxisomal matrix protein import is inhibited during mitosis.^48^
  c. Centrifuge the tubes in an SW 55 Ti swinging-bucket rotor for 15 min at 12,100 × *g* and 16°C, then place the tubes on ice (Figure 3B, step 3).
    ***Note:*** The crush spin will separate the eggs' contents into three fractions: a yellow lipid-rich layer on top; a straw-colored cytoplasmic layer (the egg extract) in the middle; and a dark pellet on the bottom consisting of pigment granules, nuclei, and other dense debris (Figure 3B, step 3).
    ***Note:*** If an SW 55 Ti swinging-bucket rotor is not available, the older SW 50.1 Ti rotor will also work.

9. Isolate the cytoplasmic fraction (Figure 4):
  a. Wipe one side of the tube with a kimwipe soaked in 100% ethanol to remove oils and other contaminants present on the outside.
  b. Pierce the cleaned side of the tube with an 18-gauge beveled needle connected to a 1-mL syringe, just above the dark pellet on the bottom of the tube (Figure 4A, step 1).
    **CRITICAL:** The needle should be oriented with the beveled side facing up (i.e., toward the cytoplasmic fraction) to avoid aspirating the pellet underneath. For easy reference, the orientation of the needle can be marked on the outside of the syringe.
    **CRITICAL:** Needles narrower than 18-gauge should NOT be used to avoid shearing organelles.
    ***Note:*** The tube wall is more easily pierced if the tube is propped vertically against the side of the bench (Figure 4A, step 1).
  c. Aspirate out the cytoplasmic layer by gently sweeping the needle from side to side while slowly pulling back the plunger (Figure 4A, step 2).
    **CRITICAL:** Pulling the plunger too fast generates shear forces in the needle that can disrupt peroxisomes and other organelles. A rate that fills a 1-mL syringe in 40–60 s is sufficient.
    ***Note:*** The sweeping motion allows the cytoplasmic fraction to be uniformly removed, and reduces the risk of forming a flowstream that could disrupt the fat layer on top or the pellet on the bottom, which should remain intact (Figure 4B).
  d. Slowly expel the contents of the syringe into a microfuge tube on ice.
  e. Repeat for any remaining tubes.
    ***Note:*** We use a clean needle and syringe for each tube to avoid shearing organelles by repeated sliding of the plunger.
    **CRITICAL:** Discard used needles in an appropriate sharps-collection container.
  f. Add an appropriate volume of the 1000× stock solution of protease inhibitors, cytochalasin, and cycloheximide to each microfuge tube, and mix by inversion.
  g. Collect the extract on the bottom of the tube using a touchspin centrifuge, and keep on ice (Figure 4C).
    ***Note:*** If the extract is significantly contaminated by debris from the pellet (i.e., the extract looks gray instead of straw-colored), it can be clarified by centrifugation. Spin the tube for 5 min at 10,000 × *g* and 4°C to sediment the debris, then transfer the supernatant to a new microfuge tube on ice using a wide-bore pipette tip.
    **CRITICAL:** Egg extract should be used promptly because it will degrade over time. In general, extract kept on ice for longer than 5–6 h should be discarded.

### Depletion of endogenous PEX5

**Timing: 30 min**

Endogenous PEX5 is depleted from egg extract using agarose beads covalently conjugated to the GST-tagged PEX5-binding domain from the docking factor PEX14. The resulting PEX5-depleted extract can then be supplemented with recombinantly produced PEX5 to restore import activity. Beads conjugated to GST alone may be used as a negative control.

See depletion of PEX5 for required materials.

10. Mix the prepared 1:1 suspension of protein-conjugated agarose beads by gently vortexing.
11. Using a wide-bore pipette tip, transfer 20 μL of the bead suspension (i.e., 10 μL of beads) to a 0.5-mL microfuge tube on ice.

***Note:*** This amount of beads is generally sufficient to deplete PEX5 from 100–200 μL of extract. The exact amount of beads needed to deplete PEX5 from a given volume of egg extract will depend on the coupling efficiency, and should be empirically determined for each batch of beads.

***Note:*** When working with small volumes of beads, it is helpful to mark the desired volume on the outside of the tube as a reference.

***Note:*** Affi-Gel beads settle quickly by gravity. The suspension should be pipetted soon after mixing.

12. Wash the beads.
  a. Add 500 μL of cold XBH buffer to the tube.
  b. Close the tube and invert a few times to mix.
  c. Collect the beads by centrifugation for 1 min at 1,500 × *g* and 4°C.
  d. Aspirate off the supernatant.
  e. Repeat two more times.

***Note:*** This step removes residual non-conjugated protein that might have detached from the beads during storage. A swinging-bucket rotor (e.g., Eppendorf model S-24-11-AT) facilitates bead collection on the bottom of the tube.

13. After the last wash, briefly spin the tube on a touchspin centrifuge to collect residual buffer on the bottom of the tube.
14. Completely aspirate off the remaining supernatant.

***Note:*** This step is facilitated by using a flattened capillary pipette tip.

15. Immediately add 200 μL of egg extract using a wide-bore pipette tip.

**CRITICAL:** A wide-bore pipette tip decreases the shear force applied to the egg extract during aspiration, and thereby reduces the risk of disrupting peroxisomes and other organelles.

***Note:*** Egg extract is very viscous. To avoid air bubbles and ensure greater accuracy, extract should be transferred by reverse pipetting.^51^ This technique involves pressing the pipette plunger past the first stop and then aspirating up a larger volume of extract then intended. The actual volume is expelled by pressing on the plunger down to the first stop and no further, so that air is not blown out of the pipette tip. Excess extract that might coat the outside of the pipette tip may be wiped off using a Kimwipe. Avoid touching the Kimwipe to the orifice of the pipette tip.

16. Mix the beads into the extract by gently swirling with a fine pipette tip.
17. Invert the tube a few times by hand, then rotate for 30 min at RT.
18. Collect the extract on the bottom of the tube by briefly spinning on a touchspin centrifuge.
19. Using a wide-bore pipette tip, transfer the mixture of extract and beads into a Micro-Spin column.
20. Partially cap the Micro-Spin column and briefly spin on a touchspin centrifuge.

***Note:*** The Micro-Spin column allows the egg extract to flow through but retains the beads in the sample reservoir on top. Ensure that all of the extract has flowed through before proceeding.

**CRITICAL:** The Micro-Spin column must be loosely capped to avoid creating a vacuum during centrifugation that might prevent all of the extract from flowing through.

21. Transfer the PEX5-depleted egg extract to a microfuge tube using a wide-bore pipette tip, and keep on ice.

***Note:*** The extent of PEX5 depletion may be assessed later by immunoblotting. Boil a small aliquot (e.g., 2 μL) of the PEX5-depleted extract in a 10-fold excess of Laemmli buffer,^52^ according to conventional protocols. As a control, boil an equivalent aliquot of unmanipulated extract. Prepare a few serial two-fold dilutions of each sample, resolve them by reducing sodium dodecyl sulfate (SDS) polyacrylamide-gel electrophoresis (SDS-PAGE), and immunoblot for PEX5 as described previously.^1^^,^^36^ Depletion efficiency may then be estimated from the resulting blot by densitometry.^53^

***Note:*** It is faster and more meaningful to assess depletion efficiency by performing a matrix protein import assay with the PEX5-depleted extract, as described in the next section. Successful PEX5 depletion should preclude import, as discussed in the expected outcomes. Once a batch of beads is standardized, this control can be included in every assay to confirm that depletion is still effective. Consult Problem 5 in the troubleshooting section for tips on improving depletion efficiency.

### Setting up an import reaction

**Timing: 30 min**

PEX5-depleted egg extract is supplemented with purified recombinant PEX5, and import is initiated by adding the fluorescent cargo GFP-SKL (reaction #1). We recommend to include two additional control reactions. First, a negative control reaction (reaction #2) using just PEX5-depleted egg extract (without recombinant PEX5) to verify the efficiency of PEX5 depletion. Second, a positive control reaction (reaction #3) using unmanipulated or mock-depleted egg extract to confirm that the peroxisomal import pathway is functional in this particular batch.

Reactions are prepared in two stages. First, all reagents **EXCEPT** the cargo are mixed in a tube and the reactions briefly warmed up. Then, import is initiated by adding cargo to the pre-warmed reactions.

See Setting up an import reaction for required materials.

22. Prepare three 0.2-mL tubes (i.e., PCR tubes) on ice.
23. To each tube, add the reagents specified in the following table **EXCEPT** GFP-SKL:

**Table:** 

Reagent	Reaction			Final concentration
	1	2	3	
PEX5-depleted egg extract	18 μL	18 μL	–	
unmanipulated egg extract	–	–	18 μL	
purified recombinant PEX5 in XBHS buffer (2 μM)	1 μL	–	–	0.1 μM
XBHS buffer	–	1 μL	1 μL	
GFP-SKL in XBHS buffer (10 μM)	1 μL	1 μL	1 μL	0.5 μM
	20 μL	20 μL	20 μL

***Note:*** The reagents should be combined in the order specified in the table, starting with egg extract. The fluorescent cargo (GFP-SKL) will be added later, after warming up the extract.

***Note:*** Dilute the purified PEX5 and GFP-SKL to the indicated 20× stock concentrations using cold XBHS buffer and keep on ice.

***Note:*** PEX5 may be used at higher concentrations (≤ 1 μM) to accelerate import,^1^ but the recommended concentration is sufficient and close to the concentration of endogenous PEX5 (∼50 nM).^54^ Higher concentrations of GFP-SKL are not recommended because they will increase the background and reduce the visibility of GFP-SKL imported into peroxisomes.

***Note:*** Egg extract is very viscous. To avoid air bubbles and ensure greater accuracy, extract should be transferred by reverse pipetting, as described above.

**CRITICAL:** To avoid shearing forces that could disrupt organelles, transfer the egg extract using a wide-bore pipette tip. Commercial wide-bore tips are usually too wide for the small volumes of extract required. We recommend cutting off a 2-mm portion of a generic tip using a clean razor blade.

24. Gently flick the tubes to mix, then collect the contents on the bottom of the tubes by briefly spinning on a touchspin centrifuge.
25. Pre-warm the tubes for 5 min at 24°C on a pre-heated 96-well thermoblock in a thermomixer, or in a block heater capable of accommodating PCR tubes.
26. Remove the tubes from the thermoblock and add the fluorescent cargo to initiate import.
27. Gently flick the tubes to mix, and then collect the contents by a brief touchspin.
28. Return the tubes to the thermoblock and incubate for 1 h at 24°C.

***Note:*** The amount of cargo imported into peroxisomes increases linearly over time.^36^ A 1-h incubation is sufficient to visualize import and detect differences between samples. Incubations longer than several hours are not recommended because of the risk of triggering apoptosis.^55^

***Optional:*** Reactions may be performed at 16°C–18ºC, which is closer to the frogs' physiological body temperature. However, egg extract becomes more viscous at lower temperatures and may be difficult to pipet. We have seen no difference in import competence between the two temperatures within the timeframe of the assay, except that import is slightly faster at 24°C.

### Preparing egg extract for imaging

**Timing: 10 min**

Each import reaction is sandwiched between two passivated coverslips and mounted on a slide using Valap sealant. While import will continue to occur during this stage of the protocol, the amount of imported cargo increases linearly with time for several hours.^36^ As long as this stage of the protocol is performed expeditiously and all samples are treated similarly, we have not noticed any effect on the results. If desired, import may be stopped by spiking each reaction with the purified PEX5-binding domain from PEX14 to a final concentration of 10 μM (which will sequester PEX5 in the cytosol and preclude further rounds of import).^36^

See imaging the import reactions for a list of required materials.

29. Prepare coverslips:
  a. Fill a clean container (e.g., a lid from a pipette-tip box) with 100% ethanol.
  b. Place the container on a block heater or a hot plate warmed to 100°C.
  c. Place 3 passivated coverslip sandwiches (one sandwich for each import reaction) into the hot ethanol bath and incubate for 5–10 min to soften.
    ***Note:*** Freshly prepared sandwiches soften within a few minutes. Sandwiches that have been stored for a week or more may require a longer incubation.
  d. Using fine-tipped tweezers, remove a coverslip sandwich from the ethanol bath.
  e. Gently pry apart the coverslips using a clean razor blade.
  f. Place the separated coverslips on a coverslip rack, ensuring that the passivated (PEGylated) surfaces are oriented in the same direction.
  g. Repeat for the remaining sandwiches.
  h. Rinse the coverslips well with 100% ethanol to remove residual PEG (immobilize the coverslips by pressing along one edge with the index finger as shown in Figure 2A).
  i. Rinse the coverslips extensively with ultrapure water.
  j. Blow off excess water with compressed air or a handheld rubber bellows.
  k. Place the rack on a hot plate warmed to 100°C until the coverslips are dry.
  l. Transfer the rack to a closed container to protect from dust, and allow the coverslips to cool to RT.
    ***Note:*** Coverslips may be prepared in advance, e.g., while the import reactions are incubating.

30. Prepare slides:
  a. Wipe 3 aluminum or glass slides with 100% ethanol to remove residual oils.
  b. Blow off any dust.

**Figure 5:**
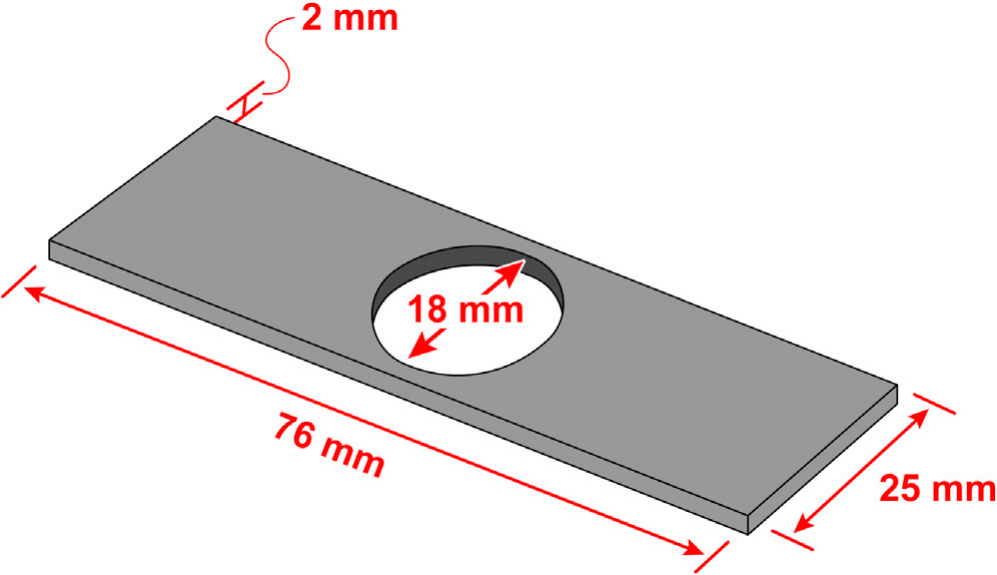
Dimensions of aluminum slides used for imaging import reactions We manufacture these in-house by abrasive waterjet cutting. The round hole reduces reflection of excitation light during episcopic fluorescence imaging, and allows the sample to be viewed diascopically by transmitted light.

***Note:*** We use aluminum slides because they are more robust and can be reused. See Figure 5 for manufacturing instructions, and step 39 for instructions on reusing aluminum slides.

***Note:*** Slides may be prepared in advance, e.g., while the import reactions are incubating.

**Figure 6:**
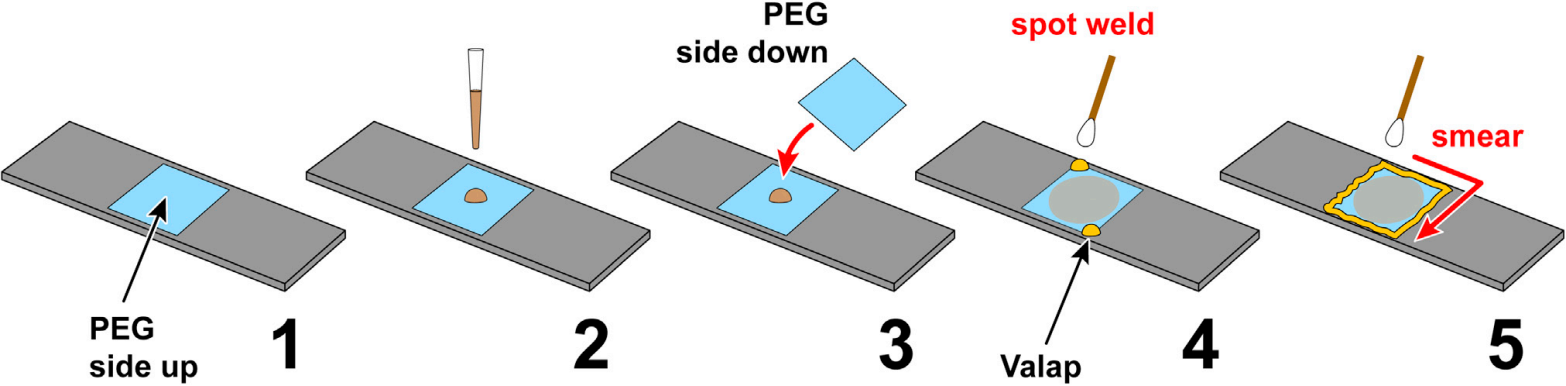
Preparation of import reactions for imaging (1) A PEGylated coverslip is placed PEG side up on a slide as shown. (2) The import reaction is spotted onto the coverslip. (3) A second PEGylated coverslip is placed PEG side down over the import reaction to form a sandwich. (4) Two corners of the sandwich are spot-welded with Valap sealant using a cotton swab. (5) The sandwich is sealed by smearing Valap around all four edges.

31. Assemble the imaging chambers (Figure 6):
  a. Using fine-tipped tweezers, gently place a clean and dry passivated coverslip in the center of a slide (Figure 6, step 1).
    **CRITICAL:** The passivated (PEGylated) side should face up.
  b. Mix the import reaction by gently flicking the tube.
    ***Note:*** Egg extract sets over time. Flicking the tube resuspends the extract to facilitate pipetting.
  c. Briefly spin down the tube on a touchspin centrifuge.
  d. Pipette 10 μL of the import reaction onto the center of the coverslip (Figure 6, step 2).
    ***Note:*** To avoid air bubbles, the import reaction should be spotted onto the coverslip by reverse pipetting, as described above. A wide-bore pipette tip is recommended.
  e. Place a second coverslip on top to form a sandwich (Figure 6, step 3).
    **CRITICAL:** The passivated side of this second coverslip should face down toward the sample.
    ***Note:*** To minimize air bubbles, it is helpful to lower the second coverslip on top of the first at an angle by starting from one side of the sandwich, instead of straight down.
    **CRITICAL:** Place the second coverslip down promptly. Waiting too long after spotting the import reaction may compromise the ability of the extract to spread evenly over the glass.
  f. Using a cotton swab, dab a little Valap sealant onto two corners of the assembled coverslip sandwich to spot-weld it to the slide (Figure 6, step 4).
  g. Immerse the cotton swab in more Valap sealant, quickly shake off any excess, and smear along one side of the sandwich to form a seal (Figure 6, step 5).
    ***Note:*** The cotton swab spreads the sealant better if it is allowed to soak in the Valap for at least several hours or even several days. We routinely reuse the same cotton swab, which can be left in the same container as the sealant and then reheated along with it.
  h. Repeat for the remaining three sides (Figure 6, step 5).
    ***Note:*** Ensure that the sandwich is completely sealed. If any gaps are present, dab with a little additional Valap.
  i. Label the slide with a marker and lay flat, with the coverslips facing up, in a slide tray to protect from light.
  j. Repeat for the remaining import reactions.

### Imaging the import reactions

**Timing: 30 min**

The assembled slides are mounted on a fluorescence microscope, and the fluorescence of the reporter cargo (i.e., GFP-SKL) is imaged at multiple positions.

**Figure 7:**
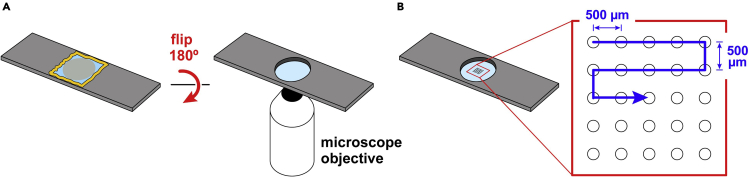
Imaging the import reactions (A) The prepared imaging chamber is mounted on a fluorescence microscope, with the coverslip side facing the microscope objective as shown. (B) Multiple fields, arrayed around the center of the coverslip and spaced at least 500 μm apart to avoid overlapping illumination, are then imaged sequentially. Blue arrow indicates the recommended imaging direction.

***Note:*** On a research-grade fluorescence microscope, this usually means that the sandwich should face downward.

32. Mount the first prepared slide in a slide holder on the microscope stage, with the coverslip sandwich facing the objective (Figure 7A).

**CRITICAL:** Handle the slides gently and move the microscope stage slowly to avoid shaking the extract. Extract that has been shaken will flow within the imaging chamber and be difficult to image.

***Note:*** Because egg extract is a uniform suspension, out-of-focus light is a considerably greater problem than when imaging intact cells. To mitigate this issue, we highly recommend using a spinning disk confocal microscope instead of a widefield setup to reduce the illuminated volume.

***Note:*** Extract cannot be easily fixed, so peroxisomes and other organelles are constantly buffeted by Brownian motion. A spinning disk confocal microscope offers a sufficiently small illuminated volume to minimize out-of-focus light, while allowing fast acquisition rates to prevent blurring. A point-scanning confocal might be too slow, despite the advantage in blocking out-of-focus light.

33. Configure the excitation light and optical elements in the microscope to be compatible with mEGFP fluorescence.

***Note:*** The spectral properties of mEGFP^43^ (and other fluorescent proteins) can be found in FPbase.^56^ As a reference, the optical elements that we use can be found in Skowyra and Rapoport.^1^

34. Bring into focus the coverslip facing the objective.

***Note:*** This step is facilitated by using an adaptive focusing system, e.g., the Nikon Perfect Focus System (PFS), the Olympus Z-drift compensation (ZDC) module, or the Leica Adaptive Focus Control (AFC). Otherwise, the coverslip must be focused manually. Manual focusing is facilitated by noting the increase in overall brightness as the objective nears the coverslip.

***Note:*** Peroxisomes in egg extract are diffraction-limited. We therefore use high-power 60× or 100× oil-immersion objectives, with a high numerical aperture (NA) of 1.4 or greater, to maximize spatial resolution and fluorescence detection. Ensure that the chosen objective is corrected for chromatic aberration and flat-field illumination (e.g., a plan apochromat objective). Again, a detailed description of the optical elements in our imaging platform can be found in Skowyra and Rapoport.^1^

35. Adjust the focus to ∼5 μm above the coverslip (i.e., toward the middle of the sandwich).

**Figure 8:**
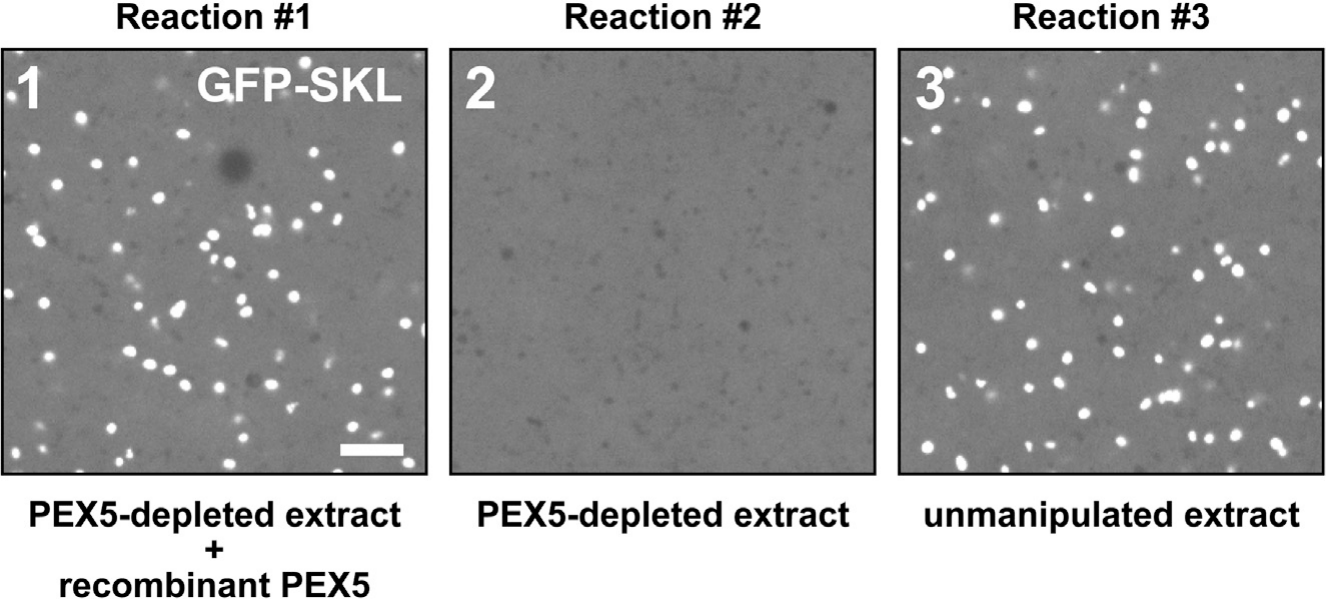
Expected outcomes of the peroxisomal import reactions Imported GFP-SKL molecules show up as bright puncta (panels 1 and 3), whereas unimported GFP-SKL produces a dimmer diffuse signal in the background (panel 2). Dark voids are vesicular compartments that exclude cytosol. Scale bar represents 5 μm.

***Note:*** If using an adaptive focusing system, adjust the offset value to reach the desired height above the coverslip. Otherwise, focus must be adjusted manually. Properly focused egg extract looks diffuse with round voids (Figure 8, panel 2), which correspond to different types of vesicular compartments.

36. Configure the acquisition software to image multiple fields arrayed around the center of the coverslip (Figure 7B).

***Note:*** We generally image a 5 × 5 or a 6 × 6 array, with individual fields separated from each other by 500 μm. This spacing yields a sufficient number of images for analysis, yet minimizes phototoxicity and photobleaching that might arise from overlapping illumination.

**CRITICAL:** Reduce the stage speed to avoid shaking the extract during acquisition.

37. Adjust the imaging parameters to maximize the signal-to-noise ratio (SNR) while avoiding saturation of the camera.

***Note:*** Pixel binning (e.g., 2 × 2) may be required to maximize the SNR. Binning should not greatly compromise spatial resolution if high-power objectives are used.

38. Acquire images and repeat for the remaining samples.
39. Aluminum slides (if using) can be cleaned and reused:
  a. Briefly heat a slide on top of a hot plate to melt the Valap sealant.
  b. Scrape off the coverslip.
  c. Wipe off the remaining sealant with a paper towel.
  d. Residual grease can be cleaned by wiping with ethanol.

**CRITICAL:** Discard used coverslips in an appropriate sharps-collection container.

## Expected outcomes

Figure 8 shows representative outcomes from each of the three import reactions. GFP-SKL that had been imported into peroxisomes will show up as bright, diffraction-limited puncta throughout the egg extract (panel 1), which represent GFP-SKL molecules concentrated inside individual peroxisomes.^36^ Unimported GFP-SKL molecules remaining in the cytosol will instead produce a much dimmer, uniformly diffuse fluorescence background (panel 2).

A successful import reaction (reactions 1 and 3) will produce both types of fluorescence patterns, because only a fraction of the total GFP-SKL molecules are imported into peroxisomes within the time frame of the assay. However, only the diffuse signal should be visible when import is blocked, e.g., by depleting PEX5 from the egg extract (reaction 2). Please see problems 2–6 in the troubleshooting section for commonly encountered problems and recommended solutions.

## Quantification and statistical analysis

A detailed procedure for normalizing images and quantifying import activity is provided in the STAR Methods section of Skowyra and Rapoport,^1^ which anyone familiar with basic functions in ImageJ^45^ should be able to implement.

## Limitations

The depletion and reconstitution procedures described in this protocol can only be used to study PEX5. However, they should be easily adaptable to other soluble components of the matrix protein import pathway that include the cargo adapter PEX7 (which recognizes a specific subset of peroxisomal proteins containing an N-terminal targeting signal), the PEX1-PEX6 ATPase, and the ubiquitination machinery. These components can be manipulated as long as suitable antibodies or binding factors are available with which to perform depletions, and the corresponding proteins can be produced recombinantly. Currently, this methodology does not allow membrane components of the matrix protein import pathway (e.g., PEX13 and PEX14, or the PEX2-PEX10-PEX12 ubiquitin ligase complex) to be depleted from egg extract and replaced with recombinant versions.

To be visible under the fluorescence microscope, GFP-SKL molecules inside peroxisomes must be numerous enough to exceed the concentration of unimported GFP-SKL in the cytosol. Otherwise, imported molecules will be indistinguishable from background. Low import activities are therefore not easily assessed using the described imaging-based protocol. To overcome this limitation, a peroxisome-impermeable GFP-quenching nanobody may be added to the reactions prior to imaging, as described previously,^36^ in order to decrease the fluorescence of unimported GFP-SKL in the cytosol. Alternatively, import may be assessed by immunoblotting for cargo after fractionating the egg extract into peroxisomal and cytosolic fractions as described in Skowyra and Rapoport.^1^

## Troubleshooting

### Problem 1

During preparation of egg extract, dejellying of the eggs takes significantly longer than 10–15 min (step 6e).

### Potential solution

• Ensure that cysteine was added to the dejellying buffer just before use and at the correct concentration.
• Ensure that the cysteine is not too old.
• Ensure that all of the cysteine has dissolved before using the dejellying buffer.
• Ensure that the final pH of the dejellying buffer is between 7 and 8.
• Gently swirling the eggs during the dejellying step is important to help the jelly coats slide off.

### Problem 2

Egg extract looks dark under the fluorescence microscope (step 38).

### Potential solution

• The fluorescent cargo (GFP-SKL) was not added to the reactions, or was added at a concentration that is too low. Ensure that the cargo is added (step 26) at a concentration of around 0.5 μM. If a different fluorescent protein is used other than GFP, the concentration might need to be optimized to account for differences in brightness.
• Imaging parameters have not been properly configured (step 33). Ensure that all filters and optical elements are compatible with mEGFP fluorescence. Try increasing the excitation light intensity or camera exposure time. Increasing pixel binning might also help, although binning greater than two-fold is not recommended due to the decrease in spatial resolution.
• Egg extract is not properly focused (step 35). If using an adaptive focusing system, the focus might sometimes lock at the wrong height. It is prudent to note the absolute height of the objective, once the extract has been properly focused, for future reference. Alternatively, the extract can be focused manually using transmitted light. In this case, look for small granular objects that correspond to vesicles and other membrane-bound compartments.

### Problem 3

Fluorescence from the cargo looks uniformly diffuse, and does not show up as bright puncta (step 38).

### Potential solution

• This is the expected result after import is blocked, e.g., by depleting endogenous PEX5. However, if no puncta are visible in the positive control reaction (i.e., in the absence of any perturbation), then it is possible that the egg extract was inappropriately prepared. Confirm that cycloheximide was added during the crush spin (step 8) to lock the egg extract in interphase, as peroxisomal matrix protein import is inhibited during cell division.^48^
• Peroxisomes might also have been lost or damaged, or an endogenous component(s) required for import might have been degraded. Ensure that all centrifugation speeds and durations specified in the protocol are strictly adhered to, and that extract is manipulated gently. Otherwise, lysosomal proteases might be released or damage to mitochondria might trigger apoptosis.^55^ It is also critical that all damaged eggs are discarded (step 5), because the contents of damaged eggs might induce apoptosis in the final extract.

### Problem 4

Supplementing PEX5-depleted extract with recombinant PEX5 does not restore import activity (step 38).

### Potential solution

• Ensure that the correct concentration of PEX5 was added to the reactions (step 23).
• Optimize the purification procedure, as PEX5 proteins are sensitive to proteolysis. Follow the procedure exactly as described in this protocol, making sure to work quickly and to keep all reagents cold, and that protease inhibitors are added during purification. Verify that the final purified protein corresponds to the right molecular weight on a gel or by mass spectrometry.
• The beads used to deplete endogenous PEX5 from egg extract were not washed sufficiently. The final wash in acidic glycine buffer is critical as it removes residual non-covalently bound protein from the resin, which might otherwise leach into the extract and inhibit import. Ensure that no protein comes off the resin after the final wash. If necessary, perform additional short washes with the acidic buffer until the flow-through runs clear.

### Problem 5

After depleting extract of endogenous PEX5, the fluorescent cargo still accumulates in bright puncta (step 38).

### Potential solution

• Depletion was insufficient, perhaps because the coupling efficiency of the PEX5-binding domain from PEX14 to the resin might have been too low. Ensure that fresh resin is used, and that the resin is washed quickly using cold water and buffer to reduce inactivation by hydrolysis. In addition, ensure that a large excess of protein to resin is used during the coupling reaction, and let the reaction proceed to completion overnight.
• An insufficient amount of resin was used to perform the depletion (step 11). Increase the amount of resin while keeping the volume of extract constant, and ensure that the resin is mixed well into the extract before starting the incubation. In addition, ensure that the extract sloshes around in the tube so that the resin can also move around, and that the depletion is performed at RT for at least 20 min to allow most PEX5 molecules to bind. If necessary, larger volumes of resin and extract might need to be used to ensure sufficient mixing.

### Problem 6

Egg extract flows in the imaging chamber and is difficult to image (step 38).

### Potential solution

• The prepared slides were handled too abruptly. Make sure to manipulate the slides gently, and avoid dropping the slides or hitting them against other objects. Also, make sure to move the microscope stage slowly once a slide has been mounted. Consult the user manual of the microscope or of the acquistion software for instructions on reducing the stage speed.
• The coverslip sandwich is not sealed completely with Valap sealant (step 31h). Gaps in the seal may cause slow evaporation of water from the extract and thereby create a flowstream.
• The coverslip sandwich is not level with the slide. Make sure that the slides have been cleaned (step 30), and that any debris has been blown off with a handheld bellows or compressed air.
• The coverslips are not sandwiched evenly. Once the passivated coverslips have been washed and dried (step 29), keep them protected from dust in the air. Residual dust can be removed with a quick puff of compressed air before assembling the sandwich.

## Resource availability

### Lead contact

Further information and requests for resources and reagents should be directed to and will be fulfilled by the lead contact, Tom A. Rapoport (tom_rapoport@hms.harvard.edu).

### Materials availability

All unique reagents generated by this study are available from the lead contact with a completed Materials Transfer Agreement.

## Data Availability

This paper does not report original code. Fluorescence micrographs were analyzed using routine functions in ImageJ. Any additional information required to reanalyze the data reported in this paper is available from the lead contact upon request.
